# How to indicate implantable cardioverter‐defibrillator in the aging population

**DOI:** 10.1002/joa3.70038

**Published:** 2025-03-13

**Authors:** Risako Orita, Naoya Kataoka, Teruhiko Imamura

**Affiliations:** ^1^ Second Department of Internal Medicine University of Toyama Toyama Japan

To Editor,

The optimal indication for implantable cardioverter‐defibrillator (ICD) implantation in the aging population remains a subject of debate, given the high incidence of nonarrhythmic mortality in this cohort. The authors have investigated the clinical outcomes of octogenarians undergoing ICD implantation for both primary and secondary prevention, with a focus on ICD therapies and the timing of mortality.^1^ Their findings suggest that while device utilization was infrequent, it preceded mortality by a significant margin. This may encourage clinicians to adopt a more aggressive approach to ICD implantation, even in elderly patients. However, several concerns warrant consideration.

A prior large‐scale study evaluating the clinical implications of ICD generator replacement in the aging population reported that a substantial proportion of patients over 80 years of age succumbed before experiencing appropriate device utilization.^2^ The discrepancy between these findings may stem from differences in baseline patient characteristics. Could the authors provide data on the proportion of patients who received guideline‐directed medical therapy, which is known to mitigate arrhythmic events? Additionally, how many patients underwent catheter ablation for ventricular arrhythmias? Given that aggressive catheter ablation can reduce arrhythmic burden and thereby decrease the need for ICD intervention,^3^ this information would be critical for contextualizing the study's findings.

Furthermore, the exclusion of patients with an observation period of fewer than 30 days raises concerns,^1^ as these individuals may be at particularly high risk for arrhythmic events. Their omission could potentially bias the results and limit the generalizability of the study.

In the present study, approximately 20% of patients received cardiac resynchronization therapy (CRT),^1^ which promotes cardiac reverse remodeling and may reduce the incidence of ventricular arrhythmias. The impact of CRT on preventing appropriate ICD utilization likely differs from that observed in patients with ICD implantation alone. Clarification on this point would enhance the interpretation of the findings.

Finally, the risk of device‐related complications, including bleeding and infection, remains a significant concern, particularly in elderly patients with multiple comorbidities.^4^ These risks must be carefully weighed against the potential benefits of ICD implantation in this population.

## CONFLICT OF INTEREST STATEMENT

The authors declare no conflict of interest.

## Data Availability

The manuscript does not include any original data.
